# Impact Buffering Characteristics of One-Dimensional Elastic–Plastic Composite Granular Chain

**DOI:** 10.3390/ma16031282

**Published:** 2023-02-02

**Authors:** Shunyuan Mao, Hui Wang, Xiaomao Wu, Huiling Zhang

**Affiliations:** School of Physical and Mathematical Sciences, Nanjing Tech University, Nanjing 211816, China

**Keywords:** granular chain, elastic–plastic deformation, energy dissipation, wave velocity, time delay

## Abstract

Considering the elastic–plastic deformation, the wave propagations and energy transmissions of the one-dimensional three-segment composite granular chain are studied. The axial symmetry model for elastic-perfectly plastic materials is built by using the finite element method. Six materials with different yield strengths are selected for the adjustable segment. The results show that the repeated loading and unloading behaviors, as well as the wave propagations in the elastic–plastic granular chain, are complex and significantly different from those in the purely elastic granular chain. The yield strength of the granular materials in the adjustable segment has significant effects on energy dissipation and wave velocity, which could be used to design the impact buffer. The studies show that taking lower yield strength for the adjustable part than the non-adjustable part, the energy dissipation could be increased, and the wave velocity could be reduced, then the arrival time of the impact waves could be delayed. These characteristics of the elastic–plastic granular chain could be used to design metamaterials for impact absorbers in impact protection.

## 1. Introduction

Impact phenomena often occur in nature and engineering applications [1,2,3], such as earthquakes, explosions, vehicle collisions, etc. These impact phenomena will not only lead to the destruction of buildings but also damage human life and health [4,5]. Therefore, how to carry out impact protection is an important content in the research of impact problems [6,7,8]. According to the mechanism of action, there are two common ways of impact protection: the first way is to confine the impact energy to the acceptable range of the impacted object [9]; the second way is to reduce the speed of impact wave propagation and extend the time of impact wave reaching the impacted object so that the impacted object has enough time to respond to the impacts [10]. 

Granular material consists of closely ordered particles in contact with each other, which has unique wave propagation characteristics and has potential applications in impact energy harvesting and mitigation [11], impact damper [12], non-destructive test [13], switches [14] and actuating devices [15]. In 1983, Nesterenko first presented the concept of granular materials and showed the highly nonlinear solitary waves in granular materials [16]. From then on, more and more attention has been paid to granular materials. As the waves in granular materials propagate through the contact of neighboring granules, the properties of the granules and the contact interactions are widely studied. 

The elastic interaction without plastic deformations is widely studied in 1D granular chains [17]. Li [18] assembled chains of elastic cylindrical granules oriented at a variable angle with respect to each other and analyzed the effect of static precompression, alignment angles, and granules’ eccentricity on the signal’s transmissibility. The results showed that cylindrical granular chain has excellent tunability and can be used as passive, tunable acoustic filtering devices and vibration absorbers. Ngo et al. studied the dynamic response of elastic 1D granular chains of uniform hollow spheres [19] and ellipsoidal granules [20]. They found that the geometry of the granules could affect wave propagation and dissipation. Khatri studied the waves in chains of elastic cylindrical granules and found that the orientation angle between the granules could be used to tune the wave propagation [21]. 

For influences of materials on wave propagations in elastic granular chains, Boechler et al. [22] studied the granular chain of the steel and aluminum alternating granules and found the modulational instability and discrete breathers. Daraio et al. [23] studied the wave propagations in PTFE granular chains and found that the waves propagate at a very low speed. Daraio [24] also found that stainless steel granules under the same static precompression force demonstrate a higher absolute increase of the solitary wave speed compared to the PTFE system through experiments and numerical calculations. This feature can be used to make tunable acoustic focusing lenses.

Sen [25] and Rosas [26] used theoretical, numerical, and experimental methods to investigate the regulation of solitary waves in 1D composite elastic granular chains from granule size, density, Young’s modulus, and arrangement, respectively. These methods could give granular chains that can be engineered with impact wave amplitudes and custom wave velocities. Huang [27] adopted the method of molecular dynamics and formed an energy trap by changing the mass ratio of granules in the heavy-light composite granular chain so as to realize the attenuation of the propagation velocity of the solitary wave and achieve the effect of impact delay. Chen [28] et al. studied the periodic binary granular chain using the binary collision approximation theory, and the impact delay can be realized by reducing the radius of the granule at the periodic position.

The applications of granular chains are not only in low-velocity impact but also in high-velocity impact, where plastic deformation or even structural damage could occur. For these inelastic cases, plastic deformations happen once the stress reaches the yield strength, and the residual deformations remain after the impact. Therefore, the energy dissipation due to plastic deformations cannot be neglected. Investigations have shown that wave propagations and contact behaviors in elastic–plastic granular chains are different from elastic ones [29,30]. The crucial interactions between the elastic–plastic granules have been widely studied by using analytical methods and finite element analysis. Stronge [31] analyzed the contact process of the elastic–plastic granules and proposed a widely used contact model, which divided the loading process into the elastic loading phase, the elastic–plastic loading phase, and the fully plastic loading phase. Pal [29] presented the force-displacement in detail by using a nonlinear finite element analysis to simulate the wave propagations in elastic–plastic chains.

On [32] studied the elastic–plastic loading conditions in the homogeneous brass granular chain through the split Hopkinson pressure bar (SHPB) experiment and found that the plastic deformation of granules under high-speed impact leads to the formation of plastic waves with slower wave speeds in the granular chains. Feng [33] studied the fluctuation characteristics caused by multiple impacts and compression processes between single contact points using the SHPB experiment and numerical simulation based on the elastic–plastic contact model [34], where the results revealed the adjustability of the impact energy dissipation. By adding individually adjustable granules at different locations in a homogeneous elastic–plastic granular chain, Pal [35] and On [36] tuned the ratios of pressure peaks, leading wave velocities, local contacts, and total dissipation at each contact point along the chain direction [29]. Burgoyne [37] used the discrete element method as well as SHPB experiments to study the homogeneous and binary elastic–plastic long chains. The results showed that the active control of impact wave propagation speed under high-speed impact could be realized by changing granule material properties, and the wave speed gradually decreased with the decrease of contact stiffness, which improves the impact delay effect.

Compared with the conventional granule arrangement, the impact buffering effect of the segmented composite granule chain is more significant. Daraio [38] experimentally captured weak separation pulses in the soft granule segments of the low elastic modulus in the multi-segment granular chain. Zhang and Xu [39,40] used experimental and numerical simulation methods to create staggered composite granular chains of materials to attenuate impact wave amplitudes and design equivalent waves [41]. Wang [42] studied the energy decay caused by the transitional behavior in the light granule region of a one-dimensional three-segment composite granular chain by means of the momentum conservation principle. Through experiments and numerical simulation, Wang [43] and Wu [44] found that increasing the length of the light chain of the three-segment granular chain can cause more energy dissipation of the granule chain as a whole.

Compared with 1D granular chains, more complex problems such as rotation, friction, tangential force, boundary configurations, and so on should be considered in 2D and 3D granular systems [45,46]. Therefore, the study of 2D and 3D granular systems is complicated. However, the properties of 1D granular chains can be scaled to predict the behaviors of 2D and 3D granular systems [47] and simplify the computational cost [48]. Therefore, the segmented composite 1D granular chains are selected as the study emphasis in this study, especially the propagation characteristics and energy dissipation mechanism under the influence of elastic–plastic deformations. By changing the material properties of adjustable-segment granules, the influences of yield strength on the energy dissipation and impact wave propagation process in the elastic–plastic granular chain are obtained, and the impact buffering characteristics are analyzed. The research results in this paper could provide a theoretical basis for energy buffering under high-speed impact conditions.

The rest of the paper is organized as follows. The finite element model for the 1D elastic–plastic granular chains is presented in Section 2. The results of the wave propagations and the influences of impact buffering are discussed in Section 3. Finally, the conclusions are drawn in Section 4.

## 2. Finite Element Model

**Figure 1:**
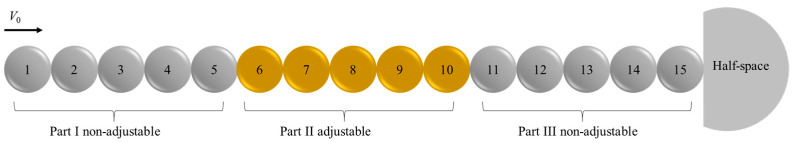
Schematic diagram of the one-dimensional three-segment composite granular chain.

In this section, LS-DYNA is used to build the finite element model of the three-segment composite granular chains. As shown in Figure 1, granule 1 impacts the three-segment composite granular chain with the initial horizontal velocity *V*_0_, where *V*_0_ is equal to the free fall velocity of height *H*. In this paper, *V*_0_ = 4.45 m/s (i.e., *H* = 1 m) is taken for research. The details of the FE model are described as follows.

### 2.1. Geometry and Material Properties

The horizontal one-dimensional three-segment composite granular chain consists of three segments, where Part I and Part III are non-adjustable segments, and the granule materials are AISI1045. Part II is an adjustable segment to investigate the effects of different yield strengths of granule materials on impact wave propagations. Each part consists of five spherical granules with the same diameter *D* (*D* = 20 mm) and material properties. The last granular is in contact with the half-space, where the material is AISI1045, and the radius of the half-space is set as 10*D* to neglect the influence of boundaries [49].

As shown in Table 1, six elastic–plastic materials with different yield strengths are selected to study the effect of yield strength on energy dissipation in the middle Part II segment, i.e., 30CrMnTi, AISI304, AISI1045, AISI4340, 10Cr2Mo1, and 40CrMnSiMoV. All six materials listed in Table 1 have similar material properties except the yield strength (see Appendix A). Therefore, to quantitatively study the influence of yield strength on impact buffering, the properties of the elastic modulus, the density as well as the Poisson ratio of all materials are set to be the same with AISI1045 material, i.e., the elastic modulus *E* = 200 GPa, the Poisson ratio *ν =* 0.3, and the density *ρ* = 7800 kg/m^3^. In order to compare the yield strength of granular materials between Part I/ Part III and Part II clearly, the yield strength ratio *λ* is defined,

(1) $$\lambda =\frac{\sigma_{Y-II}}{\sigma_{Y-I}}$$

where $\sigma_{Y-I}$ is the yield strength of Part I and Part III, $\sigma_{Y-II}$ is the yield strength of Part II.

### 2.2. Configurations and Convergency Analysis

The material type 3 in LS-DYNA, i.e., the *MAT_PLASTIC_KINEMATIC model, is suited to model isotropic and kinematic hardening plasticity. By setting the tangent modulus in material type 3 as zero, the elastic-perfectly plastic model is used for the following simulations. The element formulation 14, i.e., the 2D axisymmetric element type, is used to build the 2D axisymmetric model of the 1D granular chain. The interactions between granule and granule, as well as the interaction between the granule and the half-space, are modeled with *CONTACT_2D_AUTOMATIC_SURFACE_TO_SURFACE. The constraint for the axisymmetric model is the displacement constraints of the outer boundary of the half-space.

**Figure 2:**
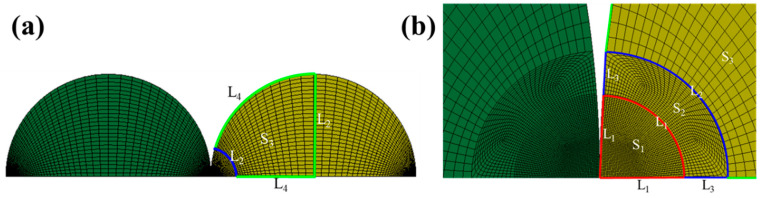
Finite element mesh of the granules: (**a**) meshes of two adjacent granules; (**b**) locally refined meshes in the contact areas.

Figure 2 shows details of the mesh in the granules, where Figure 2a plots the mesh of two adjacent granules, and Figure 2b shows the locally refined meshes in the contact areas. In Figure 2b, the whole area of one granule is divided into three areas to ensure the accuracy as well as the efficiency of the mesh. Area S_1_ is a sector area with a radius of *r*, and S_2_ is the sector ring with a radius of 1.5*r* adjacent to S_1_, where *r* is the contact radius. Area S_3_ is the remaining area of the granule. To ensure the quality of the mesh, the three edges of area S_1_ are divided by the same mesh number L_1_ and the mesh number L_2_ = 0.5L_1_ for the S_2_ outer radius. Area S_3_ is meshed with a 0.1 progressive scale.

The convergence analysis is carried out for two adjacent granules. Table 2 shows the mesh convergence results. As shown in the table, when the number of meshes in the dense area within the contact radius (L_1_) increases from 50 to 200, the minimum element size goes from 28.90 μm to 7.51 μm, the total elements increase from 109,980 to 1,380,000, the total nodes increase from 112,755 to 1,388,115, while the maximum contact force *F****_m_*** and contact duration *tc* are almost unchanged. The last column in Table 2 shows the total computation times *T*, where the computations are running in a computer with 32 processors of AMD R9 3950X (RAM: 64 GB, main frequency: 3.5 GHz). It can be seen that as the total elements increase, the computational efficiency decreases greatly. Therefore, to ensure the effectiveness of finite element analysis and computation efficiency, 80 meshes within the contact radius are selected for the modeling in this paper.

The key to successfully modeling the FE model of 1D granular chains is to determine the actual contact radius *r*, which needs several trial computations. For the first trial, a rough contact radius *r* is obtained through a rough FE model. Then a refined FE model by setting L_1_ = 80 is built according to the mesh method shown in Figure 2. After 1–2 times of refinement and simulations, the actual contact radius information can be confirmed. The actual contact radius for every contact pair of all cases is listed in Table 3, where ‘1–2’ means the contact pair between No. 1 and No. 2 granules, and so on.

## 3. Results and Discussion

In this section, the impact buffering characteristics of the 1D elastic–plastic composite granular chain is analyzed. Section 3.1 describes the wave propagations and the granular motions of elastic–plastic chains. Section 3.2 shows the elastoplastic deformation behavior of the granular chain under the influence of yield strengths. Section 3.3 analyzes the impact buffering characteristic of the three-segment composite granular chain.

### 3.1. Wave Propagations in Granular Chains

**Figure 3:**
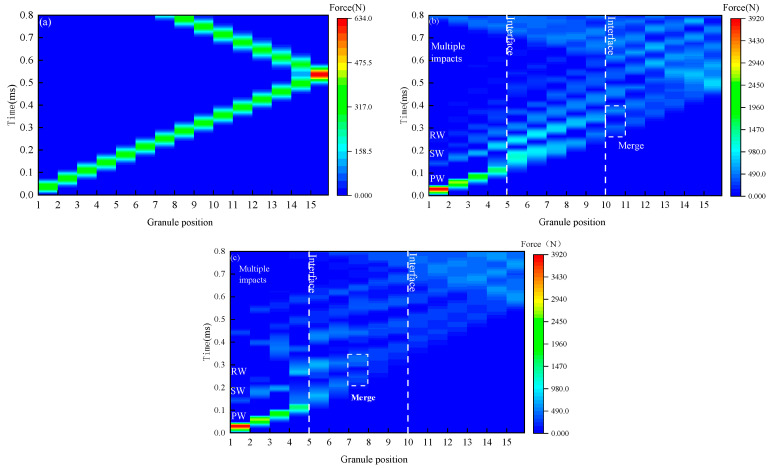
Comparison of impact wave propagation in elastic granular chains and elastic–plastic granular chains: (**a**) Homogeneous elastic granular chain (*V*_0_ = 0.45 m/s, *E* = 200 GPa, *ν* = 0.3, *ρ* = 7800 kg/m^3^); (**b**) Homogeneous elastic–plastic granular chain (*λ* = 1); (**c**) Heterogeneous elastic–plastic granular chain (*λ* = 0.23).

Figure 3 plots the wave propagations in the pure elastic granular chain and elastic–plastic granular chains. It shows that impact wave propagations could be found in granular chains. The solitary wave propagations are stable in the homogeneous elastic granular chain (see Figure 3a) with no energy decay before the impact wave reaches the interface of the last granule and the half-space, while wave propagations in elastic–plastic chains (see Figure 3a,b) appear complicated phenomena, such as the decay of the contact force, the merge of the primary wave (PW), the secondary wave (SW), and the residual wave (RW).

**Figure 4:**
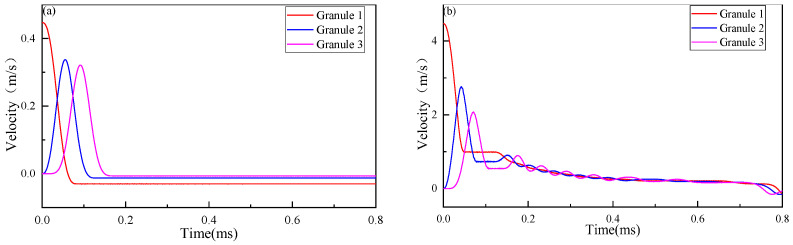
Velocity-time curves of the first three granules: (**a**) Homogeneous elastic granular chain; (**b**) Homogeneous elastic–plastic granular chain (*λ* = 1).

In order to analyze the complicated motion of the granules, the velocity-time curves of the first three granules are shown in Figure 4. Figure 4a shows that in the homogeneous elastic granular chain, granule 1 enters a state of uniform motion of −0.029 m/s at *t* = 7.7 × 10^−2^ ms, which means that granule 1 is rebounded after the contact with granule 2, and there are no further impacts. As shown in Figure 4b, in the homogeneous elastic–plastic granular chain, the maximum velocity of granule 2 is reached at *t* = 4.2 × 10^−2^ ms. In addition, granule 1 begins to move forward with a uniform velocity of 0.995 m/s at *t* = 4.9 × 10^−2^ ms, which means that granules 1 and 2 have been separated. Due to the contact between granule 2 and granule 3, the velocity of granule 2 decreases. Granule 2 begins to move at a uniform velocity of 0.730 m/s at *t* = 8.3 × 10^−2^ ms, which is smaller than the velocity of granule 1. Therefore, granule 1 catches up with granule 2 again at *t* = 1.22 × 10^−2^ ms, which causes a second impact and forms the secondary wave propagation. These complicated motions of elastic–plastic granules result in the kinetic energy of the impact granules being transferred into the granular chain several times, and the impact waves propagate in the same direction in the granular chain as there is no rebounding. This multiple-impact phenomenon results in the decomposition of the impact energy. The decompositions are marked as the primary wave (PW), the secondary wave (SW), and the residual wave (RW), which have been marked in Figure 3b.

**Figure 5:**
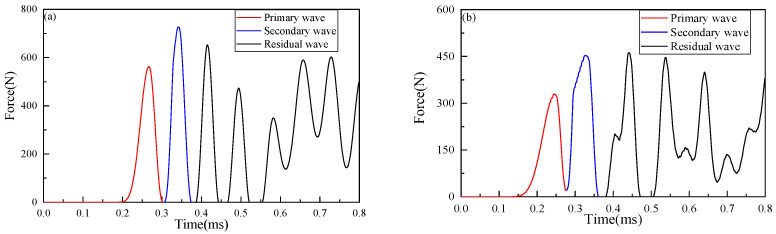
Contact force-time curves at different granule positions of Case 3 (*λ* = 1): (**a**) Granules 9 and 10 in homogeneous elastic–plastic granular chain; (**b**) Granules 7 and 8 in an inhomogeneous elastic–plastic granular chain.

As the primary wave propagates along the chain toward the half-space direction, the velocity of the primary wave decreases gradually and is smaller than that of the secondary wave. As shown in Figure 3b, the time difference between the secondary wave and the primary wave decreases significantly, in which some granules are loaded again before the end of the, and form the merging of the primary wave and the secondary wave. In order to analyze the merge of the waves, Figure 5 plots the contact force history of Case 3 (*λ* = 1), where the merge is clearly observed. As shown in Figure 5a, for impact between granules 9 and 10, the time differences of the primary wave, the second wave, and the residual wave are very small, which reveals the merge tendency. The mergence finally happened between granules 10 and 11, as marked in Figure 3b. As can be seen from the figure, the mergences could result in the peak value of the secondary wave being larger than that of the primary wave. Therefore, the impact wave will decompose, merge, and dissipate in the elastic–plastic granular chain, forming a more complex wave propagation process than in pure elastic granular chains.

For inhomogeneous elastic–plastic granular chains, Figure 3c plots the wave propagations. By comparing with Figure 3b, it can be found that when the yield strength of granule material in Part II decreases, the merging position is advanced to the contact point between granule 7 and granule 8, merging occurs in the adjustable part, and energy dissipation occurs simultaneously. As shown in Figure 5b, when *t* = 2.78 × 10^−2^ ms, the primary wave will enter the subsequent loading before it is completely unloaded. The peak value of the secondary wave is significantly larger than that of the primary wave. Therefore, the phenomenon of impact wave decomposition and mergence is more complex and significant in the inhomogeneous elastic–plastic granular chain. The high-frequency impact between granules causes high-frequency energy scattering, which leads to fast attenuation of impact energy.

### 3.2. Effect of Yield Strength on the Contact Force and Deformations

**Figure 6:**
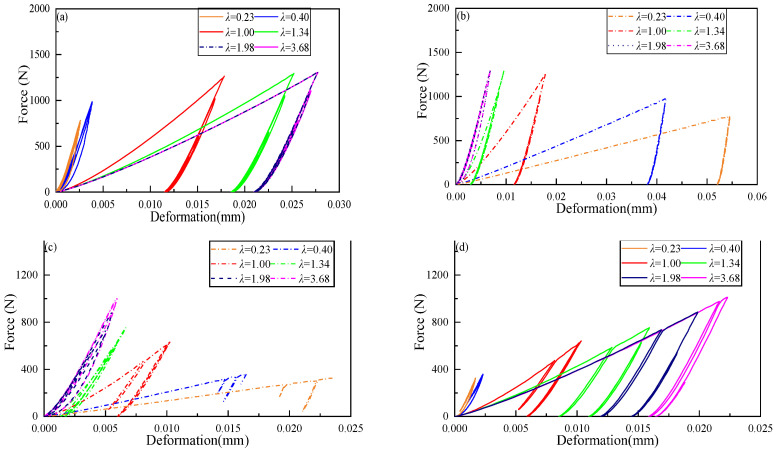
Contact force-deformation curves at different granule positions in six cases: (**a**) Contact force versus deformation of granule 5; (**b**) Contact force versus deformation of granule 6; (**c**) Contact force versus deformation of granule 10; (**d**) Contact force versus deformation of granule 11.

In order to analyze the elastic–plastic deformations, Figure 6 shows the force-deformation curve of granules 5 and 6 at the interface of Part I and Part II and granules 10 and 11 at the interface of Part II and Part III. It can be seen from the figure that when the granular chain is a homogeneous elastic–plastic granular chain (*λ* = 1), the residual deformations of granules at the interface are the same. When the granular chain is an inhomogeneous elastic–plastic granular chain (*λ* ≠ 1), the residual deformations of granules at the interface are quite different.

Table 4 shows the corresponding values of residual deformations under each case in Figure 6. In the process of numerical simulation, the yield strength of granules 5 and 11 is 530 MPa. When the yield strength ratio *λ* < 1, the residual deformations occur in lower yield strength granules 5 and 11. When *λ* = 1, the granular chain is homogeneous, and the residual deformations of granules 5 and 6 are the same, as well as the residual deformations of granules 10 and 11. With the increase of *λ*, the plastic deformations of granules 6 and 10 at the interface decrease gradually, while those of granules 5 and 11 gradually increase. When *λ* increases to a certain value, the residual deformations of granules 6 and 10 no longer occur. The above results show that for the elastic–plastic granular chain impact process considering plastic deformation, granules with relatively low yield strength are more likely to produce plastic deformations.

In order to further analyze the elastic–plastic interactions between the granules, Table 5 lists the maximum contact force *F_m_*, the maximum deformation *δ_m_*, and the residual deformation *δ_r_* for all cases. It shows that for lower *λ*, the granule deforms easily in Part II. Therefore, the *F_m_* and *δ_m_* are large. The energy dissipation could be analyzed from the variation of *δ_r_*; that is, a larger *δ_r_* represents more plastic deformation and more energy dissipation. It shows that lower *λ* could result in larger plastic deformation and more energy dissipation.

It also can be observed from the loading and unloading process in Figure 6 that the maximum contact force at the interfaces of Part I and Part II, as well as Part II and Part III, are different. The maximum contact force at the interface of Part I and Part II occurs during the first impact loading, and the subsequent loading is elastic. Therefore, the maximum contact force of the subsequent loading is smaller than that of the first loading. The maximum contact force at the interface of Part II and Part III occurs in the process of secondary loading. Since the wave velocity is proportional to the slope of the force-displacement curve [50], the slope of the elastic reloading part is higher than that of the plastic loading part for high-speed impact. Therefore, the secondary wave with sufficient amplitude travels faster than the primary wave and merges with the primary wave at a certain propagation distance. The amplitude of the corresponding wave also increases, resulting in the maximum contact force at the contact point not occurring in the initial loading.

**Figure 7:**
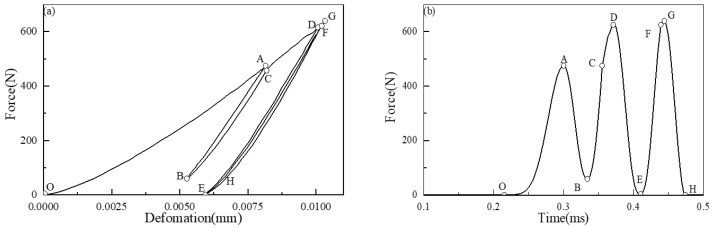
Multiple loading and unloading behaviors in Case 3 (*λ* = 1): (**a**) Contact force-deformation curve of the contact pair between granule 10 and granule 11; (**b**) Contact force-time curve of the contact pair between granule 10 and granule 11.

Figure 7 plots the multiple loading and unloading behaviors in Case 3 (*λ* = 1), which can show the elastic–plastic deformation clearly. It can be seen that within 0.5 ms, granules 10 and 11 collide three times: the OAB stage is the first impact process, where point O is the time when the impact wave arrives at the interface, the OA stage is the initial loading process, and AB stage is the initial unloading and incomplete unloading process; BCDE stage is the second impact process, in which BCD stage is the second loading process, BC stage is the elastic loading process, CD stage is the elastic–plastic loading process, and DE stage is the second unloading and incomplete unloading process; EFGH stage is the third impact process, in which EFG stage is the third loading process and merges with the second wave, EF stage is the elastic loading process, FG stage is the elastic–plastic loading process, and GH stage is the complete unloading process, that is, 10 and 11 granules begin to separate. It can be seen from Figure 3b that during the second loading stage, the primary wave is not completely unloaded, and the secondary impact enters the loading stage to form a secondary wave, which is chased and merged with the primary wave, resulting in the increase of the peak value of the wave. Therefore, point G is the maximum contact force at the interface of Part II and Part III under case 3. It can be seen from the above analysis that the contact force in the process of multiple impacts in a short time passes through the fast loading-unloading stage and causes the high-frequency fluctuation oscillation of the impact wave propagation in the granular chain.

### 3.3. Effect of Yield Strength on Impact Buffering

**Figure 8:**
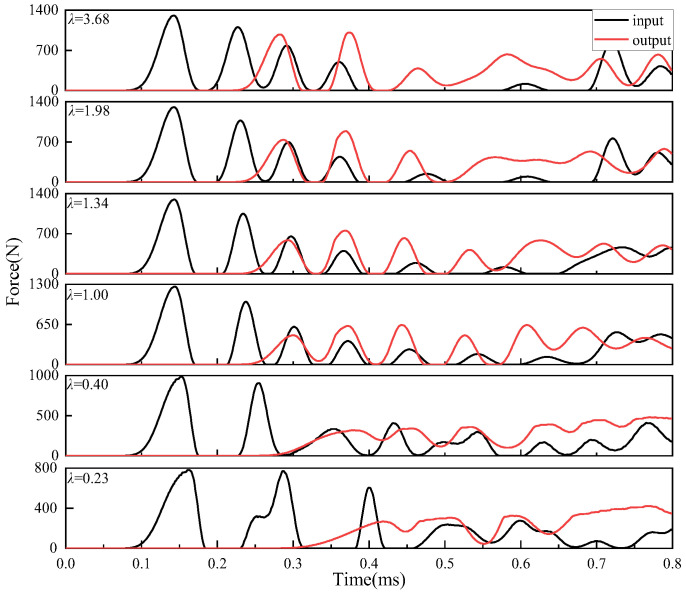
Contact force-time curves of Part II input interface and output interface under six cases.

Figure 8 shows the force-time history curve of the Part II input interface (i.e., contact interface of granule 5 and granule 6) and output interface (i.e., contact interface of granules 10 and 11). It can be seen from the figure that when the yield strength of Part II is lower than that of Part I (*λ* < 1), the maximum contact force of the Part II input interface decreases, and the corresponding output time is delayed. When the impact wave passes through Part II, the maximum contact force of the output interface of Part II decreases with the decrease of the yield strength of Part II, and the arrival time of the output wave is also delayed. When the yield strength of Part II is greater than that of Part I (*λ* > 1), the peak value of the input interface contact force increases, and the peak value appears earlier. When the impact wave propagates through Part II, the peak value of the output interface contact force of Part II increases with the increase of the yield strength of Part II, and the arrival time of the peak value of the output wave is also gradually advanced.

To intuitively analyze the energy dissipation in Part II, *η*_1_ is defined as,

(2) $$\eta_{1}=\frac{F_{m\left(10-11\right)}}{F_{m\left(5-6\right)}}$$

where $F_{m\left(5-6\right)}$ is the maximum contact force of Part II input interface, i.e., the maximum contact force between the granules 5 and 6; $F_{m\left(10-11\right)}$ is the maximum contact force of Part II output interface, i.e., the maximum contact force between the granules 10 and 11.

**Figure 9:**
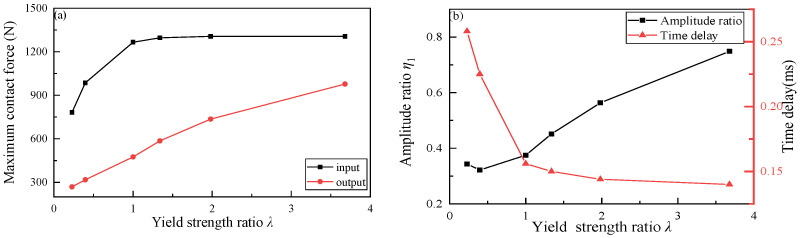
The impact buffering performance in Part II: (**a**) maximum contact force; (**b**) amplitude ratio and the time delay.

Figure 9 plots the maximum contact force, the energy dissipation, and the time delay under different yield strength ratios. As shown in Figure 9a, the higher the yield strength of Part II granules, the greater the input energy to Part II, and the greater the output energy from Part II to Part III. As shown in Figure 9b, with the increase of yield strength of Part II granules, the larger *η*_1_ is, the smaller the energy loss in Part II is, and the smaller the time delay of impact wave reaching the output interface of Part II is; on the contrary, with the decrease of yield strength of Part II granules, the time for impact wave to reach the output interface of Part II is prolonged.

**Figure 10:**
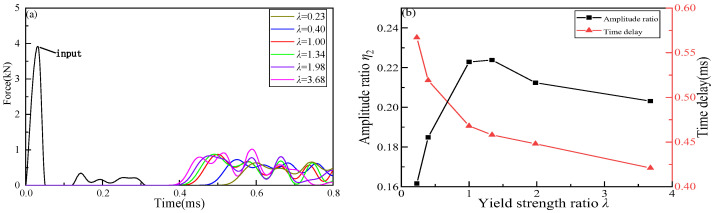
The impact buffering performance in the granular chain: (**a**) force-time history; (**b**) the energy dissipation and time delays.

In order to analyze the impact buffering performance of the whole granular chain, the impact wave propagations process of the whole granular chain under six cases is plotted in Figure 10, where Figure 10a shows the input and output impact waves of the whole granular chain and Figure 10b shows the corresponding maximum contact force ratio and output wave delay time.

To analyze the energy dissipation of the granular chain, *η*_2_ is defined as,

(3) $$\eta_{2}=\frac{F_{m\left(15-h\right)}}{F_{m\left(1-2\right)}}$$

where $F_{m\left(1-2\right)}$ is the maximum contact force between the granules 1 and 2; $F_{m\left(15-h\right)}$ is the maximum contact force between the last granule 15 and the half-space.

It can be seen from Figure 10b that when the yield strength of Part II granular material decreases, the energy dissipation increases, and the time of impact wave arriving at the output interface is delayed. When the yield strength of Part II granular material increases, the energy dissipation does not change monotonously, but the time of impact wave arriving at the output interface is always ahead of time. Furthermore, *η*_2_ increases first and then decreases with the increase of yield strength ratio *λ*, and reaches the maximum value in the range of *λ* from 1 to 1.34. In the range of *λ* < 1, with the decrease of *λ*, *η*_2_ rapidly decreases, and energy dissipation gradually increases. In the range of *λ* > 1.34, *η*_2_ also decreases with the increase of *λ*, but the absolute value of the change rate is obviously smaller than that in the range of *λ* < 1. It can be seen from Figure 9b and Table 4 that with the increase of yield strength, the impact energy obtained by Part III gradually increases, and the residual plastic deformation of granules in Part III also gradually increases, that is, the energy dissipation generated by the impact energy through plastic deformation increases, leading to the decrease of *η*_2_. To sum up, the impact waves with a high yield strength ratio in the whole granular chain are unnecessary to reduce the impact energy but can significantly reduce the arrival time of impact waves.

In order to further analyze the propagation time delay of impact wave in the granular chain, the wave velocity *v* is defined as,

(4) $$v=\frac{D}{\Delta t}$$

where *D* is the diameter of the granule, and Δ*t* is the initial response time difference of contact force between adjacent granules.

**Figure 11:**
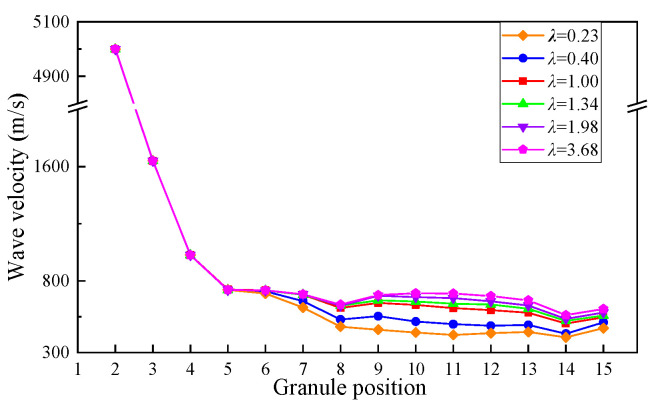
Wave velocity in the whole granular chain.

The wave velocity distributions of impact wave in the whole granular chain under six cases are shown in Figure 11. It can be seen that the wave velocity of granular chain Part I is basically the same in the six cases. The wave velocity of Part II is quite different. With the decrease in yield strength of granular materials, the wave velocity gradually decreases. This is mainly due to the difference in energy loss in the contact process caused by the different yield strengths in Part I and Part II, which ultimately leads to the difference in energy input to Part II. For elastic–plastic impacts, the impact wave velocity is determined by the impact load [50]; that is, the lower the impact load, the smaller the impact wave velocity. It can be seen from Figure 9a that when the yield strength of Part II is reduced, the impact load obtained by Part II is also lower, so the wave velocity is correspondingly reduced.

In the same way, it can be seen from Figure 9a that after the propagation of Part II, the energy input into Part III decreases with the reduction of the yield strength of Part II, so the impact wave velocity in Part III also decreases accordingly. In conclusion, when the yield strength of granules in Part II decreases, the overall impact wave propagation velocity of the granular chain decreases, and the impact wave propagation time increases. Therefore, the time delay for impact waves reaching the half-space gradually increases with the decrease of the yield strength of Part II granules.

## 4. Conclusions

A one-dimensional three-segment elastic–plastic composite granular chain analysis model is established by using the finite element method. The influences of yield strength on the energy dissipation and the arrival time of impact wave in a granular chain under high-velocity impact are studied. The results show that the elastic–plastic composite granular chain is able to tune the amplitude and wave velocity by adjusting the yield strength of granules. The main conclusions are as follows:

(1) The complex repeated loading and unloading behaviors in the elastic–plastic composite granular chain are obviously different from those in the pure elastic granular chain.
(2) The yield strength of the granular material in the adjustable part could be used to tune the impact buffering with the decrease of the yield strength of granular materials in the adjustable segment (*λ* < 1), the energy dissipation of the granular chain increases, the propagation velocity of the impact wave decreases, and the time of the impact wave reaching the boundary is delayed. With the increase of yield strength of granular materials in the adjustable segment (*λ* > 1), the overall energy dissipation of the granular chain does not change monotonously, but the propagation velocity of the impact wave keeps increasing, and the time of the impact wave reaching the boundary is advanced.
(3) For the design of a high-speed impact buffering, the protection of the impacted structure/object could be achieved by reducing the yield strength of the material in the adjustable part of the elastic–plastic granular chain, which could increase the energy dissipation and delay the arrival time of the impact waves.

1D segment composite granular chain considering elastic–plastic deformation is the main work of this paper, which provides potential application in mitigation systems and impact buffering. However, further studies in complex elastic–plastic 2D and 3D granular systems are expective. To study the 2D and 3D granular systems, the rotation, tangential interaction, packing configurations, and so on should be modeled. The application based on the 1D granular chain model built in this paper to 2D and 3D granular systems is the further research work.

## Figures and Tables

**Table 1:** Yield strength of six granular materials of Part II.

Cases	Material	Yield Strength *σ_y_*/MPa	Yield Strength Ratio *λ*
Case 1	30CrMnTi	121	0.23
Case 2	AISI304	210	0.40
Case 3	AISI1045	530	1.00
Case 4	AISI4340	710	1.34
Case 5	10Cr2Mo1	1050	1.98
Case 6	40CrMnSiMoV	1950	3.68

**Table 2:** Contact convergence analysis.

*L*_1_	Δ*l*/μm	Total Elements	Total Nodes	*F_m_*/N	*tc*/ms	* T *
50	28.90	113,607	116,542	3654.08	8.61 × 10^−2^	3 h 33 min
80	18.80	248,400	252,376	3658.13	8.63 × 10^−2^	10 h 13 min
100	15.00	392,375	397,426	3630.68	8.63 × 10^−2^	17 h 46 min
200	7.51	1,467,500	1,476,566	3630.11	8.63 × 10^−2^	≈239 h

**Table 3:** The maximum contact radius *r* for all cases (mm).

Contact Pair	*r* of Case 1	*r* of Case 2	*r* of Case 3	*r* of Case 4	*r* of Case 5	*r* of Case 6
1–2	0.98	0.98	0.98	0.98	0.98	0.98
2–3	0.83	0.83	0.83	0.83	0.83	0.83
3–4	0.73	0.73	0.73	0.73	0.73	0.73
4–5	0.63	0.63	0.63	0.63	0.63	0.63
5–6	0.89	0.76	0.57	0.56	0.55	0.55
6–7	0.75	0.65	0.52	0.48	0.44	0.38
7–8	0.68	0.59	0.49	0.45	0.42	0.38
8–9	0.59	0.54	0.45	0.44	0.41	0.38
9–10	0.61	0.52	0.40	0.43	0.40	0.38
10–11	0.63	0.52	0.38	0.43	0.46	0.49
11–12	0.39	0.40	0.38	0.42	0.46	0.49
12–13	0.38	0.39	0.36	0.46	0.46	0.46
13–14	0.40	0.43	0.45	0.46	0.49	0.46
14–15	0.40	0.43	0.46	0.44	0.49	0.52
15-half space	0.45	0.48	0.53	0.51	0.50	0.50

**Table 4:** Residual deformation of granules at the interface(mm).

Cases	Granule 5	Granule 6	Granule 10	Granule 11
Case 1	0	0.053	>0.020 (Not completely unloaded)	0
Case 2	0	0.038	>0.015 (Not completely unloaded)	0
Case 3	0.012	0.012	0.0059	0.0059
Case 4	0.019	0.0028	0.0016	0.011
Case 5	0.021	0	0	0.014
Case 6	0.021	0	0	0.016

**Table 5:** Variations of *F_m_*, *δ_m_*, and *δ_r_* for all cases.

Cases	Granules 5 and 6			Granules 10 and 11		
	*F_m_*/N	*δ_m_*/mm	*δ_r_*/mm	*F_m_*/N	*δ_m_*/mm	*δ_r_*/mm
Case 1	781.75	0.057	0.053	325.26	0.025	>0.020
Case 2	985.57	0.046	0.038	359.03	0.019	>0.020
Case 3	1265.48	0.036	0.024	640.08	0.021	0.012
Case 4	1296.16	0.035	0.0218	754.17	0.023	0.013
Case 5	1305.82	0.035	0.021	885.89	0.025	0.014
Case 6	1305.67	0.035	0.021	1011.46	0.028	0.016

## Data Availability

The data presented in this study are available on request from the corresponding author.

## Appendix A

The actual properties of materials used in this paper are shown in Table A1.

**Table A1:** Material properties.

Material	Elastic Modulus *E*/GPa	Density *ρ*/(kg/m^3^)	Yield Strength *σ_y_*/MPa	Poisson’s Ratio *ν*
30CrMnTi	205	7850	121	0.250
AISI304	190	8000	210	0.290
AISI1045	200	7800	530	0.300
AISI4340	205	7850	710	0.300
10Cr2Mo1	201	7830	1050	0.291
40CrMnSiMoV	193	7780	1950	0.260
